# Mechanical colon obstruction due to the alimentary limb after Roux-en-Y gastric bypass: a case report

**DOI:** 10.1186/s13256-020-02645-w

**Published:** 2021-02-03

**Authors:** Caspar Joyce Peterson, Jennifer Klasen, Tarik Delko, Romano Schneider

**Affiliations:** Clarunis, Department of Visceral Surgery, University Center for Gastrointestinal and Liver Diseases, St. Claraspital and University Hospital Basel, 4002 Basel, Switzerland

**Keywords:** Gastric bypass, Bariatric surgery, Bowel obstruction, Colon obstruction, Alimentary limb, Case report

## Abstract

**Background:**

Small bowel obstruction is a known and potentially lethal complication after gastric bypass surgery, in both the early and the late postoperative course. Colon or large bowel obstruction, on the other hand, seems to be rare after gastric bypass surgery and thus is not routinely considered.

**Case presentation:**

We present the case of a 21-year old morbidly obese caucasian patient who underwent laparoscopic Roux-en-Y gastric bypass surgery and developed an early severe transverse colon obstruction due to compression of the transverse colon by the antecolic alimentary limb. Emergency revisional surgery showed a short and tense alimentary limb mesentery and possibly tight closure of Petersen’s space contributing to the compression. Through opening of Petersen’s space and mobilization of alimentary limb mesentery, decompression was achieved, and the patient fully recovered.

**Conclusions:**

This is a rare case of colon obstruction caused by direct compression of the transverse colon by the antecolic alimentary limb. We propose that a combination of short tense alimentary limb mesentery and perhaps tight closure of Petersen’s space was responsible for the obstruction in this case. Surgeons and treating physicians need to be aware of such rare causes of early postoperative bowel obstruction and take these into consideration when evaluating patients.

## Background

With morbid obesity on the rise worldwide, the number of patients undergoing gastric bypass procedures is steadily increasing. Such operations warrant thorough peri- and postoperative care as well as regular follow-up because of various associated complications. While small bowel obstruction (SBO) is a known and potentially severe complication in both the early and late postoperative course after Roux-en-Y gastric bypass (RYGB) surgery, colonic obstruction seems to be rare [1–3]. Early postoperative SBO is most often caused by kinking at the jejuno-jejunostomy and results in subsequent obstruction of the bypass with life-threatening dilatation of the remnant stomach [4, 5]. This risk of kinking is slightly higher with closure of mesenteric defects and can be reduced by applying an anti-kinking (anti-obstructive) stitch at the jejuno-jejunostomy [4–6]. Late postoperative SBO occurs mostly due to internal hernia (IH) through the mesenteric defects (jejuno-mesenterial and Petersen’s space) created during the RYGB procedure [2, 3]. The incidence of IH after laparoscopic gastric bypass surgery is highest 1–2 years postoperatively, corresponding to the time of greatest weight loss [4, 7, 8]. However, in both the early and late postoperative course after gastric bypass surgery, reports of mechanical colon obstructions of any etiology are rare.

## Case presentation

**Fig. 1:**
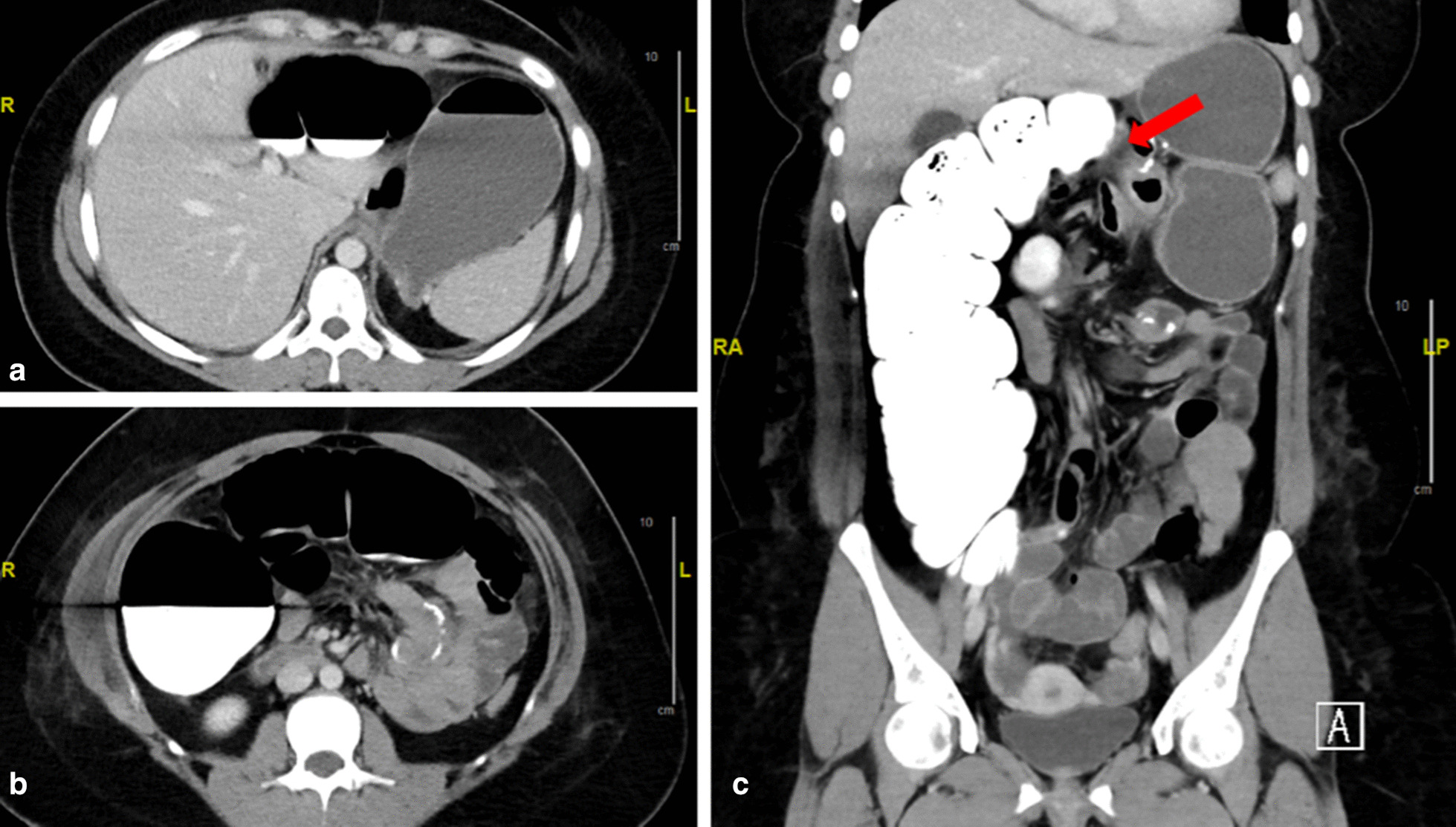
Abdominal computed tomography scan on the fourth postoperative day showing signs of colon obstruction. **a** Dilated and fluid-filled remnant stomach. **b** Dilated right hemicolon, normal presentation of gastro-jejunostomy. **c** Dilated right hemicolon with change of caliber, normal presentation of jejuno-jejunostomy. Red arrow points to the antecolic alimentary limb compressing transverse colon

A 21-year old morbidly obese caucasian female patient was referred to our bariatric center for surgical treatment. Patient history revealed she had undergone a laparoscopic fenestration of an ovarian cyst 7 years prior without complications. The body mass index (BMI) at the time of consultation was 36.8 kg/m^2^ (84 kg, 151 cm). Standardized diagnostic workup included a pulmonary and cardiologic check-up, and an ultrasound of the gallbladder and liver, which revealed a mild liver steatosis and no further anomalies. A gastroscopy, which had been performed 6 months earlier because of dyspepsia, showed a small (1 cm) axial hiatal hernia with grade A reflux esophagitis and negative biopsies for *Helicobacter pylori* [9]. After consulting the patient and discussing the case in our interdisciplinary bariatric team, the decision was made to perform a laparoscopic RYGB procedure [10]. The patient provided informed consent. At the time of surgery, diagnostic laparoscopy showed a larger than expected hiatal hernia with transposition of the gastroesophageal junction 3–4 cm into the thoracic cavity. Thus, after repositioning the stomach into the abdominal cavity, a posterior cruroraphy with braided nonabsorbable single sutures was performed. Next, a laparoscopic RYGB with antecolic Roux-en-Y limb [alimentary limb (AL)] was performed. No further irregularities were encountered during surgery. The gastro-enterostomy was hand-sewn with a running suture using monofilament absorbable synthetic sutures, and a linear stapler was used to perform the jejuno-jejunostomy. Monofilament nonabsorbable polypropylene sutures were used for closure of all mesenteric defects (jejuno-mesenterial and Petersen’s space) with single stiches. The upper gastrointestinal X-ray series performed routinely on the first postoperative day showed regular postoperative results with no leakage or signs of obstruction. Thus, a diet consisting of liquids and mushy foods was started. On the same day the patient complained of mild abdominal pain in the right upper quadrant and reduced appetite. The patient did not have symptoms of nausea or vomiting. The cardiopulmonary parameters and body temperature were always normal. On the fourth postoperative day the patient complained of sudden nausea, increasing abdominal pain and fullness. The abdomen was distended, and an emergency computed tomography (CT) scan was obtained. The CT scan showed an obstruction of the transverse colon with a sudden change of caliber dorso-caudally to the stomach proximally to the jejuno-jejunostomy, and distension of right transverse and ascending colon including cecum (Fig. 1). A fluid-filled small bowel and remnant stomach were seen as signs of intestinal congestion, while the gastric pouch, the gastro-enterostomy and the jejuno-jejunostomy displayed regularly. Consequently, the patient was taken into the operating theater for an emergency laparoscopy. Because of massive abdominal distension resulting in too little space for pneumoperitoneum, conversion to laparotomy had to be made. Exploration showed compression of the transverse colon by the antecolic AL and subsequent massive distension of the ascending and right transverse colon. Through opening of the closed Petersen’s space and mesenteric mobilization of the AL, the pressure on the transverse colon was released. Because of the massively distended colon and compromised colonic wall with threatening perforation, a decompressing colotomy was performed. Three serosal tears in the ascending colon, caused by the massive distension, were sutured. As per local protocol in early postoperative bowel obstruction after RYGB surgery, a percutaneous endoscopic gastrostomy (PEG) tube was placed in the remnant stomach to ensure lasting decompression and provide monitoring. Subsequently, the abdominal fascia was closed, and a subcutaneous negative-pressure wound therapy (NPWT) device was installed to ease the tension on the abdominal fascia as per local protocol. Postoperatively, an antibiotic therapy with ceftriaxone and metronidazole was administered. Oral intake was limited to small amounts of clear fluid, and parenteral nutrition was initiated. One day after revisional surgery, the patient experienced tachycardia, and pulse oximetry showed peripheral oxygen saturation levels of 80% despite nasal oxygen substitution. In the subsequent thoracic CT scan, no signs of pulmonary embolism were found, while bilateral pleural effusions were seen, and therapy with diuretics was administered. Because of persistent leukocytosis and high C-reactive protein count on the seventh day after the initial operation, 3 days after the revisional surgery, a second-look laparotomy was performed. A minor leakage at the gastric entry point of the PEG tube was seen, while calibers of the colon, remnant stomach and small bowel were normal. The leakage was corrected, the abdominal fascia was closed and a subcutaneous NPWT installed. Five days later, secondary closure of the laparotomy wound was achieved and the further postoperative course was uneventful. Antibiotic therapy and parenteral nutrition were eventually stopped, and the PEG tube closed off as full oral intake was possible. Twenty-two days postoperatively and 18 days after revisional surgery for obstruction, the patient was discharged in good condition.

Three weeks after discharge during a scheduled follow-up in the outpatient clinic, the PEG tube was removed and the patient had no complaints. Weight loss of 15 kg was documented at that time. Six months postoperatively the patient was again seen in the outpatient clinic, where she complained of postprandial abdominal pain and intermittent odynophagia. An ultrasound of the gallbladder and a gastroscopy showed no signs of gallstones and gastric or anastomotic ulcer, respectively, and symptomatic therapy was started. One-year follow-up showed a weight rebound to 80 kg (BMI 35.1 kg/m^2^) and lasting nonspecific abdominal pain. A gastrogastric fistula between the gastric pouch and the remnant stomach as possible cause for the rebound was excluded via upper gastrointestinal (GI) series and a gastroscopy. At the time of writing this report, therapy with a glucagon-like peptide-1 (GLP-1) agonist is being started in addition to existing dietary measures and exercise, and the patient remains in regular follow-up.

## Discussion and conclusions

Several complications are associated with gastric bypass surgery. Early surgical complications, such as anastomotic leakage, can lead to peritonitis. However, typical clinical signs of peritonitis are absent in the obese patient, thus warranting close postoperative monitoring in this population. Nonspecific signs such as fever spikes, tachycardia and hiccups should prompt aggressive diagnostic measures in these patients. If tachycardia is present, intra-abdominal pathology and pulmonary embolism must be actively excluded by imaging studies [1]. Late surgical complications such as anastomotic fistula and anastomotic stenosis may develop several months after gastric bypass surgery [11]. Anastomotic stenosis typically affects the gastro-jejunostomy, leading to regurgitation due to the obstruction [12]. The most common cause of early postoperative bowel obstruction is kinking at the jejuno-jejunostomy, which we primarily suspected in our case. Early and late obstruction mostly affects the small bowel, and reports of colon or large bowel obstruction are scarce in the current literature. Rehfuss *et al.* reported a case of cecal volvulus caused by IH through the mesocolic defect 13 years after open RYGB surgery with retrocolic AL [13]. The patient in that case had the RYGB surgery in 2003, and it is unclear whether the mesenteric defects were closed during surgery. However, we may assume that the closure of mesenteric defects was not routinely done in 2003. With the switch from open to laparoscopic RYGB, an increase in the incidence of IH was observed, mostly attributed to fewer adhesions in laparoscopic than open surgery. Nowadays, effective measures to reduce the risk of IH after laparoscopic gastric bypass surgery include antecolic AL placement and closure of all mesenteric defects during surgery with nonabsorbable sutures. Closure of mesenteric defects has been shown to significantly decrease the incidence of IH and subsequent SBO, and a RYGB with antecolic AL and closure of both jejuno-mesenterial and Petersen’s space results in the lowest rate of IH [4, 14, 15]. Thus, in our bariatric surgery center, we routinely close all mesenteric defects. Closure of jejuno-mesenterial space is usually easily done. More caution needs to be applied when closing Petersen’s space, not to puncture or ligate the middle colic artery, which lies in direct anatomical proximity. Our patient underwent a laparoscopic RYGB with antecolic AL, closure of all mesenteric defects by nonabsorbable sutures and placement of an anti-kinking stitch. Opening of the closed Petersen’s space and mobilization of the short and tense AL mesentery during revisional surgery eased the pressure on the transverse colon. Because of the massive distension of the colon with threatening perforation, it was decided intraoperatively to do a controlled colotomy instead of dealing with the risk of subsequent perforation. A decompressing colostomy was discussed as an alternative to the colotomy. However, ostomy surgery in obese patients can pose technical difficulties due to the thick layer of subcutaneous fat, and obese patients are at a higher risk of ostomy-related complications [16].

We propose that tight closure of Petersen’s space with resulting tension to an anatomically short antecolic AL mesentery was responsible for the obstruction. In assuming a low incidence of transverse colon compression by the AL, while an open Petersen’s space would in turn significantly increase the risk of IH and late postoperative SBO, we encourage the continued practice of closing all mesenteric defects. However, it is important that surgeons and treating physicians are aware of cases like the one presented and take them into consideration in the peri- and postoperative course.

## Data Availability

All data generated or analyzed during this study are included in this published article. Additional information is available from the corresponding author upon reasonable request.

## Abbreviations

SBO: Small bowel obstruction
RYGB: Roux-en-Y gastric bypass
IH: Internal hernia
BMI: Body mass index
AL: Alimentary limb
CT: Computed tomography
PEG: Percutaneous endoscopic gastrostomy
NPWT: Negative-pressure wound therapy
GLP-1: Glucagon-like peptide-1

## References

[1] Kassir R, Debs T, Blanc P, Gugenheim J, Ben Amor I, Boutet C, *et al.* Complications of bariatric surgery: presentation and emergency management. Int J Surg. 2016;27:77–81. 10.1016/j.ijsu.2016.01.067 26808323

[2] Higa K, Ho T, Tercero F, Yunus T, Boone KB. Laparoscopic Roux-en-Y gastric bypass: 10-year follow-up. Surg Obes Relat Dis. 2011;7(4):516–25. 10.1016/j.soard.2010.10.019. 21333610

[3] Abasbassi M, Pottel H, Deylgat B, Vansteenkiste F, Van Rooy F, Devriendt D, *et al*. Small bowel obstruction after antecolic antegastric laparoscopic Roux-en-Y gastric bypass without division of small bowel mesentery: a single-centre, 7-year review. Obes Surg. 2011;21(12):1822–7. 10.1007/s11695-011-0462-6. 21656166

[4] Stenberg E, Szabo E, Ågren G, Ottosson J, Marsk R, Lönroth H, *et al.* Closure of mesenteric defects in laparoscopic gastric bypass: a multicentre, randomised, parallel, open-label trial. Lancet. 2016;387:1397–404. 10.1016/S0140-6736(15)01126-5 26895675

[5] Stenberg E, Szabo E, Ågren G, Näslund E, Boman L, Bylund A, *et al.* Early complications after laparoscopic gastric bypass surgery: results from the Scandinavian Obesity Surgery Registry. Ann Surg. 2014;260(6):1040–7. 10.1097/SLA.0000000000000431 24374541

[6] Brolin RE. The antiobstruction stitch in stapled roux-en-Y enteroenterostomy. Am J Surg. 1995;169(3):355–7. 10.1016/S0002-9610(99)80175-5. 7879843

[7] Brolin RE, Kella VN. Impact of complete mesenteric closure on small bowel obstruction and internal mesenteric hernia after laparoscopic Roux-en-Y gastric bypass. Surg Obes Relat Dis. 2013;9(6):850–4. 10.1016/j.soard.2012.11.007. 23415691

[8] Schneider C, Cobb W, Scott J, Carbonell A, Myers K, Bour E. Rapid excess weight loss following laparoscopic gastric bypass leads to increased risk of internal hernia. Surg Endosc. 2011;25(5):1594–8. 10.1007/s00464-010-1444-9. 21072668

[9] Sami SS, Ragunath K. The Los Angeles classification of gastroesophageal reflux disease. Video J Encycl GI Endosc. 2013;1(1):103–4. 10.1016/S2212-0971(13)70046-3.

[10] Richtlinien zur operativen Behandlung von Übergewicht (Medizinische Richtlinien) Swiss Society for the Study of Morbid Obesity and Metabolic Disorders (SMOB). 2018. p. 1–31. https://www.smob.ch/de/component/jdownloads/?task=download.send&id=61&catid=3&m=0&Itemid=101.

[11] Hamilton EC, Sims TL, Hamilton TT, Mullican MA, Jones DB, Provost DA. Clinical predictors of leak after laparoscopic Roux-en-Y gastric bypass for morbid obesity. Surg Endosc. 2003;17(5):679–84. 10.1007/s00464-002-8819-5. 12618940

[12] Brethauer SA, Chand B, Schauer PR. Risks and benefits of bariatric surgery: current evidence. Clevel Clinic J Med. 2006;73(11):993–1007. 10.3949/ccjm.73.11.993. 17128540

[13] Rehfuss JP, Friedman JE, Tan SA, Lottenberg LL, Goldstein LE. Cecal volvulus caused by internal herniation after roux-en-y gastric bypass surgery. J Surg Case Rep. 2018;2018(9):rjy241. 10.1093/jscr/rjy241. PMC6131969 30214713

[14] Harakeh ABA, Kallies KJ, Borgert AJ, *et al.* Bowel obstruction rates in antecolic/antegastric versus retrocolic/retrogastric Roux limb gastric bypass : a meta-analysis. Surg Obes Relat Dis. 2016;12(1):194–8. 10.1016/j.soard.2015.02.004. 26003892

[15] Escalona A, Devaud N, Pérez G, Crovari F, Boza C, Viviani P, *et al.* Antecolic versus retrocolic alimentary limb in laparoscopic Roux-en-Y gastric bypass: a comparative study. Surg Obes Relat Dis. 2007;3(4):423–7. 10.1016/j.soard.2007.04.005. 17544923

[16] Braumann C, Müller V, Knies M, Aufmesser B, Schwenk W, Koplin G. Complications after ostomy surgery: emergencies and obese patients are at risk—data from the Berlin OStomy study (BOSS). World J Surg. 2019;43(3):751–7. 10.1007/s00268-018-4846-9. 30426187

